# Successful Outcome After Intralesional Curettage for Spindle Cell Hemangioma of Fibula in an Infant: A Case Report

**DOI:** 10.3389/fped.2021.767927

**Published:** 2021-11-04

**Authors:** Tao Han, Rufa Wang, Xiaoguang Zhou

**Affiliations:** ^1^Department of Burns and Plastic Surgery, Children's Hospital of Nanjing Medical University, Nanjing, China; ^2^Department of Orthopedic Surgery, Children's Hospital of Nanjing Medical University, Nanjing, China; ^3^Neonatal Medical Center, Children's Hospital of Nanjing Medical University, Nanjing, China

**Keywords:** spindle cell hemangioma, intralesional curettage, outcome, vascular lesions, fibula

## Abstract

Spindle cell hemangioma (SCH), a non-neoplastic reactive vascular lesion, rarely locates in bones. We herein report a successful case of intralesional curettage for an infant with SCH of fibula. An 11-month-old boy was admitted to our center with a painless mass in the right proximal calf. Preoperative digital radiograph demonstrated a massive vascular lesion with an irregular bone destruction of proximal fibula. The lesion was removed via the intralesional curettage approach and pathologically diagnosed as SCH. The patient gained bone structure recovery of right proximal fibula. Two years after the surgery, he experienced no local recurrence. For the management of SCH of fibula with partial bone destruction, we suggest early-stage intralesional curettage as its safety and effectiveness.

## Introduction

Spindle cell hemangioma (SCH), characterized by cavernous blood vessels and spindle cell proliferation, has recently been considered as a non-neoplastic reactive vascular lesion (1, 2). SCH often occurs at early age with high risk of recurrence after surgery due to its uncertain border with surrounding tissue. It commonly arises in the dermal or subcutaneous tissue of the distal extremities (3, 4). Reports on SCH cases involving bone are rare, most of which focus on histopathological description, but lack sufficient clinical and follow-up data (5, 6). Herein, we present a case of SCH in proximal fibula that was managed successfully by intralesional curettage and, moreover, discuss its clinical characteristics and long-term surgical outcome.

## Case Report

**Figure 1:**
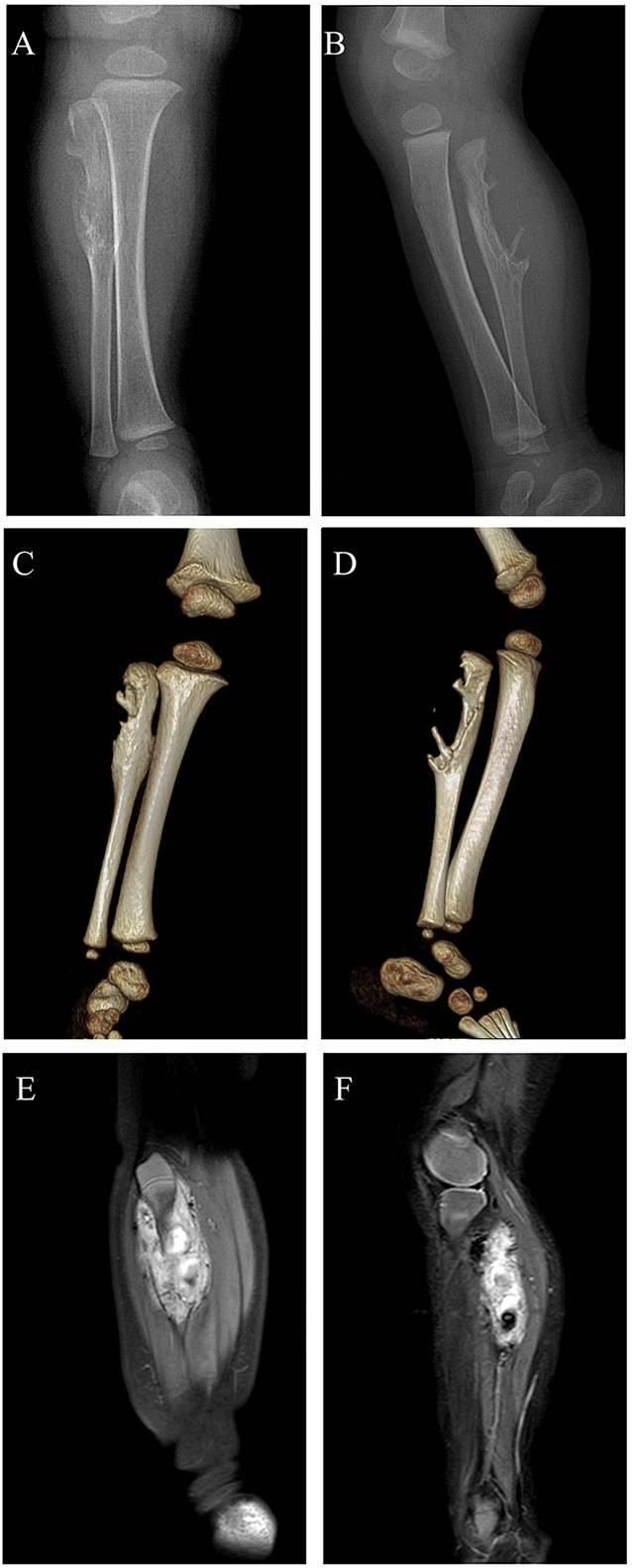
Digital radiograph preoperatively: **(A,B)** Radiographs of the right tibia and fibula showing an irregular bone destruction of proximal fibula; **(C,D)** 3d CT reconstruction demonstrating lytic bone destruction of right proximal fibula; **(E,F)** MRI revealing a massive vascular tumor with surrounding soft tissue hyperplasia and involvement of the proximal fibular epiphyseal plate.

An 11-month-old boy patient presented to our center with a 2-month history of painless mass in the right proximal calf. The mass had been noted to be slowly enlarging in 3 weeks after presentation. No significant symptom was found in this patient. Initial workup performed included radiograph, three-dimensional CT (3d CT) reconstruction, and MRI. Radiographs of the right tibia and fibula indicated an irregular bone destruction of proximal fibula (Figures 1A,B), and the lytic bone destruction was confirmed by 3d CT reconstruction (Figures 1C,D). MRI revealed a massive vascular tumor with surrounding soft tissue hyperplasia and involvement of the proximal fibular epiphyseal plate (Figures 1E,F).

**Figure 2:**
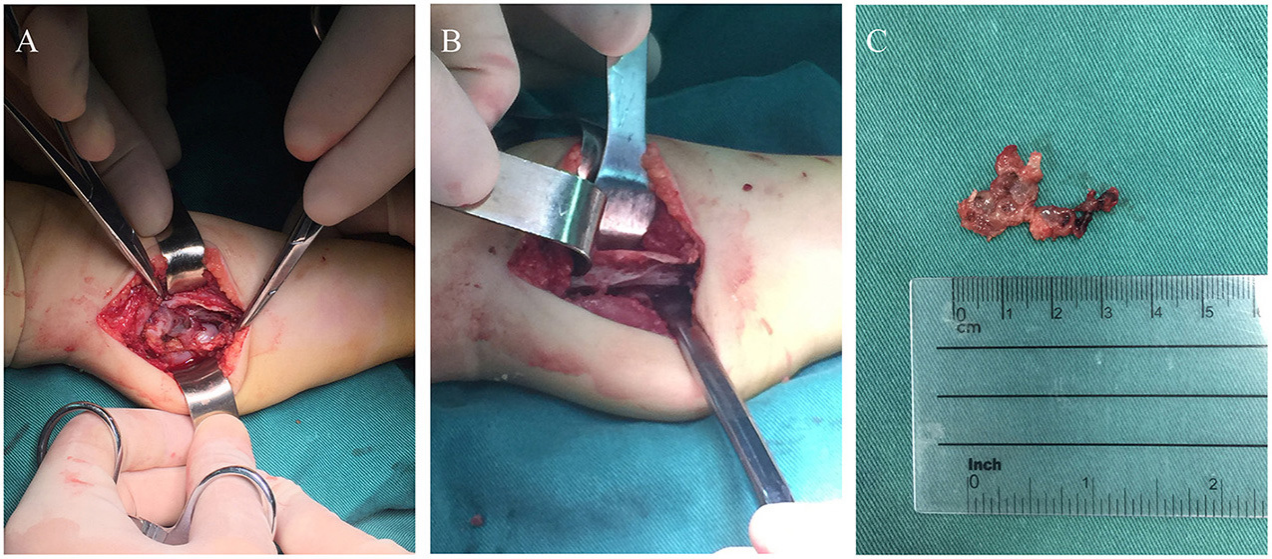
The photograph during the surgery: **(A)** Intraoperative image of the surgical finding of a vascular mass attached to proximal fibula; **(B)** Complete curettage of lesion to normal fibular surface; **(C)** Macroscopic appearance of the excised lesion.

**Figure 3:**
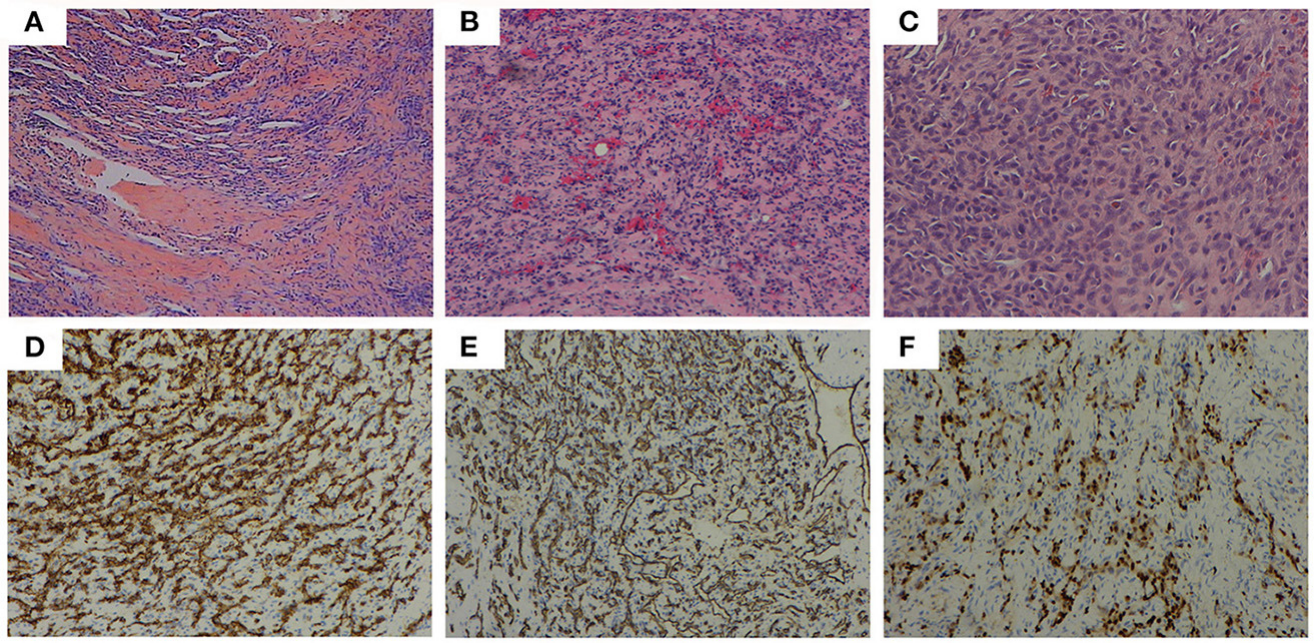
Histopathological features: **(A)** (HE, ×40) the fissure-like vessel lumens lined with flattened endothelial cells among the spindle cells, **(B)** (HE, ×100), **(C)** (HE, ×200) the spindle shaped cells arranging in fascicular pattern in solid area. Immunohistochemical analysis revealing positive staining for **(D)** CD31 (×100), **(E)** CD34 (×100), and **(F)** ERG (×100) in the majority of spindle cells.

**Figure 4:**
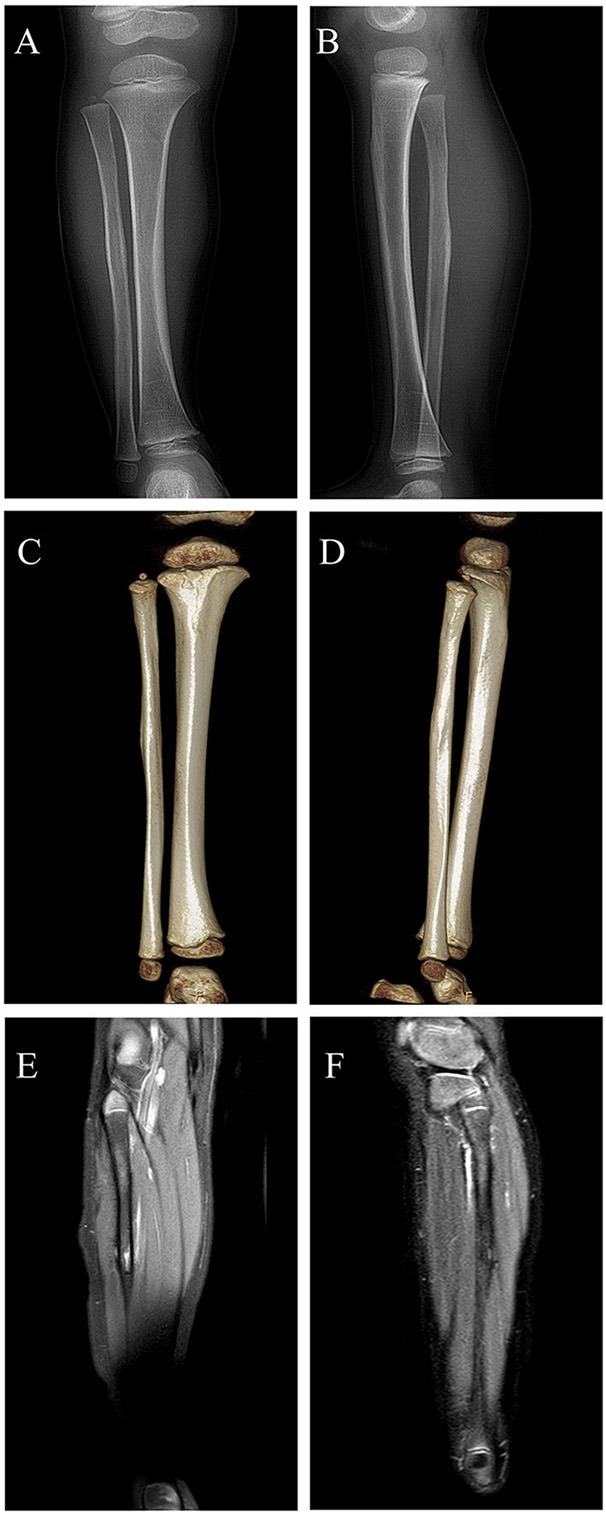
Digital radiograph at 2 years postoperatively: **(A,B)** Radiographs showing reformation of the cortex of the proximal fibula; **(C,D)** 3d CT reconstruction demonstrating both uniform bone mineral density and continuous cortical of right proximal fibula; **(E,F)** MRI revealing remarkable regression of lesion without evidence of local recurrence.

Considering partial bony structure of proximal fibula was normal, intralesional curettage was performed on the right proximal fibula under general anesthesia. After lesion exposure, a 4.02 × 0.0-cm-sized vascular mass was identified with extension to proximal fibular. Gross examination showed a reddish spongious solid mass, containing topical hemorrhage, partial thrombosis, and irregular bone destruction (Figure 2A). With protection of common peroneal nerve and peripheral vessels, complete curettage of lesion was performed to normal periosteum of fibular (Figures 2B,C). Histologically, the lesion was characterized by the fissure-like vessel lumens lined with flattened endothelial cells among the spindle cells (Figure 3A). The spindle-shaped cells arranged in fascicular pattern in solid area, with similar cell morphology and no atypia (Figures 3B,C). Immunohistochemically, the endothelial cells lining the vessel spaces stained positive for CD31, CD34, and ERG (Figures 3D–F). Therefore, with standard of international society for study of vascular anomalies (ISSVA) classification (7), the diagnosis of spindle cell hemangioma was made in this patient according to the clinical and histopathologic manifestations. On postoperative follow-up, this patient was asymptomatic without any evidence of recurrence. Two years after this surgery, he returned to hospital for outpatient review. Radiographs showed the reformation of the cortex of the proximal fibula (Figures 4A,B), and both uniform bone mineral density and continuous cortical of right proximal fibula were confirmed by 3d CT reconstruction (Figures 4C,D). Besides, MRI demonstrated remarkable regression of lesion without any signs of tumor growth through the fibula (Figures 4E,F).

## Discussion

This case is rare in comparison with majority of reported SCH cases and merits discussion on following points: location of lesion, selection of surgical intervention, histopathologic characteristics, and long-term postoperative follow-up. SCH is a benign vascular lesion which generally locates in the subcutis at the distal extremities and presents as solitary and multifocal masses. It also can be associated with several clinical syndromes, among which Maffucci syndrome is the most common (8, 9). In several uncommon cases, SCHs have been found in lips, nasal passage, temporal muscle, and even in lungs and spleen (2, 10–13). In comparison, the reported cases of SCH arising in bones are even more unusual so far (14–16). In our case, a solitary lesion of SCH involved the proximal fibula with surrounding soft tissue hyperplasia, while the superficial skin and tissues were normal.

To date, the main treatment choice for fibular tumor is segmental or subperiosteal resection, in case of local recurrence at surgical site (17–19). Wide excision for clear margin has always been first choice for SCHs, and repeated surgical resection is performed if a recurrence occurs (3, 20). However, given that preoperative digital radiograph indicated that the vascular mass on fibula was solitary, and part of both cortex and cancellous fibula were not involved, intralesional curettage was selected as the surgical intervention in this case for achieving the maximum retention of healthy bony structure. During the operation, complete curettage was performed to the normal fibular surface without residual lesion.

The histologic appearance in this case consisted of the fissure-like vessel lumens lined with flattened endothelial cells among the spindle cells, which arranged in fascicular pattern in solid area. CD31, CD34, and ERG, as vascular endothelial markers, were reported positive expression in various kinds of vascular tumors. In this case, immunohistochemical analysis revealed positive staining for CD31, CD34, and ERG in the majority of spindle cells, consistent with the diagnosis of SCH (21, 22). Metastasis of SCH is rare, although local recurrence may occur (20, 23). On the most recent imaging examination, 2 years after the initial surgery, our patient was still disease free and found to experience entire reformation of bone structure of the right proximal fibula. This indicates the safety and effectiveness of intralesional curettage for the management of this case.

In conclusion, for SCH of fibula with partial bone destruction, intralesional curettage renders a safe and efficient intervention at early stage. Meanwhile, our study was limited by lacking further evaluation for treatment approaches owing to small sample size. Long-time and consistent follow-up could establish the efficacy of our management.

## Data Availability Statement

The original contributions presented in the study are included in the article/supplementary material, further inquiries can be directed to the corresponding author.

## Ethics Statement

The studies involving human participants were reviewed and approved by the Ethics Committee of the Children's Hospital of Nanjing Medical University. Written informed consent to participate in this study was provided by the participants' legal guardian/next of kin. Written informed consent was obtained from the minor(s)' legal guardian/next of kin for the publication of any potentially identifiable images or data included in this article.

## Author Contributions

XZ revised the manuscript and approved the final manuscript as submitted. RW performed the surgery and conducted the data analyses. TH wrote sections of the article and edited the figures. TH and RW contribute to this work equally. All authors read and approved the final manuscript.

## Conflict of Interest

The authors declare that the research was conducted in the absence of any commercial or financial relationships that could be construed as a potential conflict of interest.

## Publisher's Note

All claims expressed in this article are solely those of the authors and do not necessarily represent those of their affiliated organizations, or those of the publisher, the editors and the reviewers. Any product that may be evaluated in this article, or claim that may be made by its manufacturer, is not guaranteed or endorsed by the publisher.
